# Magnesium Sulfate in Pediatric Emergency Medicine: Pharmacology, Evidence, and Clinical Applications

**DOI:** 10.7759/cureus.91672

**Published:** 2025-09-05

**Authors:** Ghaith K Mansour, Amal Yousif

**Affiliations:** 1 Pharmaceutical Sciences Department, Alfaisal University College of Pharmacy, Riyadh, SAU; 2 Emergency Department, Pediatric Emergency Unit, King Saud University Medical City, Riyadh, SAU

**Keywords:** bradyarrhythmias, life-threatening arrhythmia, pediatric seizure, preeclampsia-eclampsia, refractory seizure, status asthmaticus

## Abstract

Magnesium sulfate (MgSO₄) is a well-established therapeutic agent with extensive historical use, predominantly recognized in obstetrics. However, its critical role in pediatric emergency care remains underrecognized despite substantial clinical evidence supporting its efficacy in severe asthma exacerbations, refractory seizures, and life-threatening cardiac arrhythmias. This review aims to consolidate and critically appraise the current evidence regarding MgSO₄ pharmacologic mechanisms, clinical applications, and therapeutic impact across major pediatric emergencies, emphasizing the potential to improve morbidity and mortality outcomes through optimized utilization. A comprehensive literature synthesis was conducted, integrating mechanistic insights with clinical trial data and guideline recommendations pertaining to MgSO₄ use in pediatric seizures, status asthmaticus, and cardiac emergencies. Emerging trends, dosing strategies, safety profiles, and comparative effectiveness were analyzed to delineate best practices. MgSO₄ demonstrates distinct electrophysiological and anti-inflammatory properties, contributing to its neuroprotective, bronchodilatory, and antiarrhythmic effects in children. Rigorous clinical evidence supports MgSO₄ as a vital adjunct in refractory status epilepticus, severe asthma not responsive to first-line therapies, and torsades de pointes or certain bradyarrhythmias. Despite these benefits, substantial disparities in clinical adoption persist globally, often due to limited awareness, protocol variability, and concerns about dosing and adverse effects. MgSO₄ represents a powerful, multi-dimensional pharmacologic agent that is critically underutilized in pediatric emergency medicine. Standardized global protocols integrating MgSO₄ could markedly enhance outcomes in seizure management, critical asthma exacerbations, and cardiac arrhythmias. This synthesis advocates for reemphasizing MgSO₄ as a first-line or adjunctive intervention in relevant pediatric emergency scenarios and supports further high-quality research to refine evidence-based guidelines.

## Introduction and background

Magnesium sulfate (MgSO₄) represents one of the most profoundly underappreciated therapeutic agents in contemporary pediatric emergency medicine. Despite six decades of clinical use, it remains underutilized in pediatric emergency medicine despite robust evidence supporting its role in asthma, seizures, and arrhythmias [1]. This essential mineral compound has emerged as a cornerstone therapy for diverse pediatric emergencies, encompassing life-threatening asthma exacerbations, complex seizure disorders, and critical cardiac arrhythmias, yet remains significantly underutilized in many pediatric emergency departments worldwide [2]. The clinical significance of MgSO₄ in pediatric populations extends far beyond its traditional obstetric applications, demonstrating remarkable versatility across a broad spectrum of emergency conditions that demand immediate and effective therapeutic intervention [2].

The historical foundation of MgSO₄ therapy in pediatric medicine traces its origins to the pioneering work over 60 years ago, which first demonstrated its remarkable bronchodilatory properties in asthmatic patients, establishing the groundwork for subsequent investigations into magnesium's multifaceted therapeutic mechanisms [3]. This seminal observation catalyzed decades of research that revealed magnesium's complex interactions with cellular calcium homeostasis, smooth muscle relaxation mechanisms, and anti-inflammatory pathways, ultimately positioning it as a critical therapeutic option in pediatric emergency care [4]. The renewed interest in MgSO₄ as a therapeutic agent has been driven by mounting evidence of its efficacy across diverse pediatric emergency conditions, coupled with its favorable safety profile, cost-effectiveness, and accessibility in resource-limited settings [1].

In pediatric emergency departments globally, MgSO₄ has established itself as an indispensable second-line therapy for severe asthma exacerbations that fail to respond adequately to conventional bronchodilator and corticosteroid treatments [5]. The therapeutic rationale for MgSO₄ in pediatric asthma encompasses far more than simple bronchodilation, involving complex interactions with cellular calcium homeostasis, adenylyl cyclase activation, and inflammatory mediator inhibition that collectively contribute to improved clinical outcomes [6]. Contemporary evidence supports the use of intravenous MgSO₄ in children presenting with acute severe asthma, with multiple randomized controlled trials demonstrating significant improvements in pulmonary function parameters, reduced hospitalization rates, and decreased need for intensive care interventions [7].

The application of MgSO₄ in pediatric seizure disorders represents another critical therapeutic domain, particularly in the management of refractory status epilepticus and febrile illness-related epilepsy syndrome, where conventional antiepileptic drugs prove inadequate [8]. The neuroprotective properties of MgSO₄ have been extensively documented in both experimental and clinical settings, with evidence suggesting its ability to modulate neuronal excitability through multiple mechanisms, including calcium channel blockade, N-methyl-D-aspartate (NMDA) receptor antagonism, and preservation of blood-brain barrier integrity [9]. These mechanisms contribute to the anticonvulsant efficacy of MgSO₄ in pediatric populations, offering an alternative therapeutic approach when traditional seizure management strategies fail to achieve adequate control [8].

Cardiac applications of MgSO₄ in pediatric emergency medicine encompass an increasingly diverse range of arrhythmic conditions, including supraventricular tachycardia, junctional ectopic tachycardia, torsades de pointes, and various forms of ventricular arrhythmias [10]. The electrophysiological effects of MgSO₄ on cardiac tissue involve multiple complex mechanisms, including L-type calcium channel modulation, sodium-potassium ATPase regulation, membrane stabilization, and altered conduction velocity through specialized cardiac conduction pathways [11]. These properties make MgSO₄ particularly valuable in pediatric patients with inherited or acquired cardiac conduction abnormalities, where traditional antiarrhythmic agents may be contraindicated due to age-related pharmacokinetic considerations or may prove ineffective due to underlying pathophysiology [10].

The pediatric-specific considerations for MgSO₄ therapy encompass numerous age-related pharmacokinetic differences, developmental variations in receptor sensitivity, physiological characteristics unique to growing children, and the specialized monitoring requirements that differ significantly from adult applications [2]. These factors necessitate careful dose adjustments, specialized monitoring protocols, and pediatric-specific safety considerations that require extensive expertise in pediatric emergency medicine to implement effectively and safely [2]. The growing body of evidence supporting MgSO₄ use in pediatric populations has led to increased recognition of its therapeutic potential and gradual incorporation into clinical practice guidelines worldwide, though significant variations in adoption and implementation remain across different healthcare systems [12].

## Review

Chemistry and formulation

The chemical identity of MgSO₄ is fundamentally defined by its molecular formula, MgSO₄·7H₂O, representing the heptahydrate crystalline structure that constitutes the most commonly utilized pharmaceutical preparation in clinical practice [13]. This inorganic salt possesses a molecular weight of 246.47 g/mol in its fully hydrated form, with the anhydrous compound exhibiting a molecular weight of 120.37 g/mol, though the anhydrous form is rarely encountered in pharmaceutical applications due to its hygroscopic nature and stability considerations [14]. The structural characteristics of MgSO₄ reflect its ionic composition, wherein magnesium exists as a divalent cation (Mg²⁺) coordinated with sulfate anions (SO₄²⁻) in a complex three-dimensional crystalline lattice stabilized by extensive hydrogen bonding networks involving coordinated water molecules [15].

The crystalline structure of MgSO₄ heptahydrate exhibits remarkable complexity, with the magnesium ion occupying octahedral coordination sites surrounded by six oxygen atoms derived from both water molecules and sulfate groups [16]. The sulfate ion adopts a tetrahedral geometry with sulfur at the center surrounded by four oxygen atoms, creating a highly polar anionic species that contributes significantly to the compound's solubility and electrolytic properties [17].

Pharmaceutical formulations of MgSO₄ are available in multiple hydration states, each with distinct physicochemical properties and clinical applications. The heptahydrate form (MgSO₄·7H₂O) represents the most stable and commonly encountered preparation under standard atmospheric conditions. Each hydration state must be considered in pharmaceutical manufacturing and storage protocols [18].

The solubility characteristics of MgSO₄ demonstrate remarkable temperature dependence, with solubility increasing dramatically as temperature rises. At room temperature (20°C), MgSO₄ heptahydrate exhibits a solubility, making it highly suitable for the preparation of concentrated intravenous solutions required for clinical applications [15]. This high solubility facilitates the preparation of solutions with concentrations up to 50% w/v, though most clinical formulations utilize concentrations between 10% and 20% to optimize both therapeutic efficacy and injection tolerability [1].

The manufacturing process for pharmaceutical-grade MgSO₄ involves rigorous purification procedures to eliminate trace contaminants that could compromise patient safety or therapeutic efficacy. Quality control measures include extensive testing for heavy metals, bacterial endotoxins, pH stability, and crystal purity using advanced analytical techniques [17].

Injectable formulations of MgSO₄ require careful attention to osmolarity, pH adjustment, and sterility to ensure compatibility with intravenous administration. Standard preparations typically maintain a pH between 5.5 and 7.0, with osmolarity ranging from 1000 to 4000 mOsm/L depending on concentration, necessitating appropriate dilution or administration through central venous access for high-concentration preparations. Preservative-free formulations are mandated for pediatric applications, particularly for intrathecal or high-volume intravenous administration, requiring specialized manufacturing and packaging procedures to maintain sterility throughout the product's shelf life [1].

Pharmacological effects and mechanism of action

The pharmacological effects of MgSO₄ in pediatric emergency medicine arise from its fundamental role as the fourth most abundant cation in the human body and its participation in over 300 enzymatic reactions that govern critical cellular processes [2]. At the molecular level, magnesium ions function as essential cofactors for adenosine triphosphate (ATP) synthesis, protein synthesis, nucleic acid metabolism, and transmembrane ion transport mechanisms that are particularly crucial during periods of physiological stress encountered in emergency situations. The therapeutic mechanisms of MgSO₄ extend far beyond simple electrolyte replacement, encompassing complex interactions with calcium homeostasis, neurotransmitter release, vascular smooth muscle function, and inflammatory cascade modulation [1].

The calcium channel antagonist properties of magnesium represent one of its most clinically significant mechanisms of action in pediatric emergency care [4]. Magnesium ions compete directly with calcium for binding sites on voltage-gated calcium channels, effectively blocking calcium influx into cells and preventing the calcium-dependent processes that drive smooth muscle contraction, neurotransmitter release, and inflammatory mediator activation [19]. This mechanism is particularly relevant in pediatric asthma management, where calcium channel blockade in bronchial smooth muscle cells leads to bronchodilation, reduced airway hyperresponsiveness, and decreased inflammatory cell activation [5]. The competitive inhibition occurs at multiple calcium channel subtypes, including L-type, N-type, and T-type channels, each contributing to different aspects of the therapeutic response observed in clinical practice [19].

In the cardiovascular system, MgSO₄ exerts profound effects on cardiac electrophysiology through multiple complementary mechanisms that collectively contribute to its antiarrhythmic properties [19]. The inhibition of calcium channels in cardiac myocytes reduces calcium-dependent action potential propagation, slows conduction velocity through the atrioventricular node, and stabilizes cardiac cell membranes against aberrant electrical activity. Additionally, magnesium modulates sodium-potassium ATPase activity, which maintains proper transmembrane ion gradients essential for normal cardiac rhythm generation and conduction. These electrophysiological effects are particularly beneficial in pediatric patients with tachyarrhythmias, premature ventricular contractions, and torsades de pointes, where traditional antiarrhythmic agents may be contraindicated due to age-related considerations [10].

The neuroprotective mechanisms of MgSO₄ involve complex interactions with multiple neurotransmitter systems and cellular protective pathways that are especially relevant in pediatric seizure management [9]. Magnesium acts as a physiological antagonist at NMDA receptors, blocking glutamate-mediated excitotoxicity that contributes to seizure propagation and neuronal injury [8]. This NMDA receptor blockade is voltage-dependent and reversible, providing seizure control without the permanent receptor alterations associated with some conventional anticonvulsants [9]. Furthermore, magnesium enhances gamma-aminobutyric acid neurotransmission, the primary inhibitory neurotransmitter system in the central nervous system, thereby promoting neuronal hyperpolarization and reducing seizure susceptibility [8].

The anti-inflammatory properties of MgSO₄ represent another crucial mechanism contributing to its therapeutic efficacy across multiple pediatric emergency conditions. Magnesium deficiency has been associated with increased production of pro-inflammatory cytokines, including tumor necrosis factor-alpha, interleukin-1 beta, and interleukin-6, while magnesium supplementation reduces these inflammatory mediators and promotes resolution of inflammatory processes [9]. In pediatric asthma, this anti-inflammatory effect complements the bronchodilatory properties by reducing airway inflammation, mucus production, and epithelial damage that characterize acute exacerbations [20]. The mechanism involves modulation of nuclear factor-kappa B signaling pathways, reduction of oxidative stress through enhanced antioxidant enzyme activity, and stabilization of mast cell membranes to prevent histamine release [21].

MgSO₄ also demonstrates significant effects on nitric oxide synthesis and vascular endothelial function, mechanisms that contribute to its therapeutic benefits in conditions involving vascular dysfunction. Enhanced nitric oxide production leads to vascular smooth muscle relaxation, improved endothelial function, and reduced vascular resistance, effects that are particularly beneficial in pediatric patients with hypertensive emergencies or conditions involving compromised tissue perfusion. The vasodilatory effects occur through both endothelium-dependent and endothelium-independent mechanisms, providing therapeutic benefits even in conditions where endothelial function is compromised [19].

At the cellular level, MgSO₄ influences mitochondrial function and energy metabolism, mechanisms that become particularly important during periods of cellular stress encountered in critical illness. Magnesium is essential for optimal ATP synthesis through its role in stabilizing ATP-magnesium complexes and facilitating oxidative phosphorylation processes within mitochondria. During hypoxic or ischemic conditions common in pediatric emergencies, adequate magnesium availability helps maintain cellular energy production and prevents the cellular dysfunction that contributes to organ failure. Additionally, magnesium stabilizes ribosomal structures and facilitates protein synthesis, processes that are crucial for cellular repair and recovery following acute illness or injury [2].

Pharmacokinetics/pharmacodynamics and pharmacogenomics

The pharmacokinetic profile of MgSO₄ in pediatric populations demonstrates significant age-related variations that necessitate careful consideration of developmental pharmacology principles when designing dosing regimens for emergency care applications [22]. Following intravenous administration, MgSO₄ undergoes rapid dissociation into magnesium and sulfate ions, with the magnesium component being the primary pharmacologically active moiety responsible for therapeutic effects [23]. The distribution of magnesium in pediatric patients follows a complex multi-compartment model, with initial rapid distribution into extracellular fluid compartments followed by slower equilibration with intracellular stores and bone tissue [22]. Approximately 99% of total body magnesium exists intracellularly or bound to bone, with only 1% present in serum, making serum magnesium levels imperfect predictors of total body magnesium status and therapeutic response [23].

The volume of distribution for magnesium in pediatric patients varies significantly with age, body composition, and disease state, ranging from approximately 0.2 L/kg in neonates to 0.4 L/kg in adolescents [22]. This age-related variation reflects developmental changes in body water distribution, muscle mass, and bone mineral content that occur throughout childhood and adolescence [23]. Children with acute illness may demonstrate altered distribution characteristics due to changes in protein binding, capillary permeability, and tissue perfusion that affect magnesium uptake and distribution to target tissues [22]. These factors contribute to significant interpatient variability in pharmacokinetic parameters and emphasize the importance of individualized dosing strategies based on clinical response rather than relying solely on standard weight-based calculations [23].

Renal elimination represents the primary route of magnesium excretion, with approximately 95% of filtered magnesium normally reabsorbed through the proximal tubule, loop of Henle, and distal convoluted tubule [22].

The renal handling of magnesium in pediatric patients demonstrates significant developmental maturation, with glomerular filtration rate and tubular reabsorption capacity both increasing throughout childhood to reach adult values by approximately two years of age [23]. In healthy pediatric patients, the elimination half-life of intravenously administered magnesium ranges from four to 12 hours, depending on age, renal function, and concurrent medications that may affect renal magnesium handling [22]. However, in critically ill pediatric patients with compromised renal function, magnesium elimination may be significantly prolonged, necessitating dose adjustments and enhanced monitoring to prevent accumulation and toxicity [23].

The pharmacodynamic relationship between serum magnesium concentrations and clinical response demonstrates considerable complexity and interpatient variability in pediatric populations [22]. Therapeutic serum magnesium levels for various emergency indications range from 2.0 to 4.0 mg/dL (0.82-1.65 mmol/L), though some conditions may require higher concentrations for optimal therapeutic effect [23]. The concentration-response relationship is influenced by numerous factors, including baseline magnesium status, concurrent medications, acid-base balance, and the specific clinical condition being treated [22]. In pediatric asthma management, bronchodilatory effects typically occur at serum concentrations above 2.5 mg/dL, while anticonvulsant effects may require concentrations exceeding 3.0 mg/dL [23].

The pharmacogenomic aspects of MgSO₄ therapy in pediatric patients represent an emerging area of research with significant clinical implications for personalized medicine approaches [22]. Genetic polymorphisms affecting magnesium transport proteins, including TRPM6, TRPM7, claudin-16, and claudin-19, can significantly influence magnesium homeostasis and therapeutic response to supplementation [23]. These transport proteins are responsible for magnesium absorption in the intestine, reabsorption in the kidney, and cellular uptake in target tissues, with genetic variations potentially altering their function and contributing to interpatient variability in therapeutic response [22]. Additionally, polymorphisms in genes encoding calcium channel subunits may affect the competitive inhibition of calcium channels by magnesium, potentially influencing the magnitude and duration of therapeutic effects [23].

Population pharmacokinetic modeling studies in pediatric patients have identified several significant covariates that influence magnesium disposition and should be considered when designing individualized dosing regimens [22]. Body weight and age represent the most important covariates affecting clearance and volume of distribution, while renal function, albumin levels, and concurrent diuretic therapy also contribute significantly to pharmacokinetic variability [23]. Disease-specific factors, including severity of acute illness, degree of inflammation, and presence of fluid overload, can further modify magnesium pharmacokinetics and necessitate dosing adjustments to achieve optimal therapeutic outcomes [22]. These findings have led to the development of sophisticated dosing algorithms that incorporate multiple patient-specific factors to optimize magnesium therapy in pediatric emergency settings [23].

The relationship between magnesium pharmacokinetics and pharmacodynamics is further complicated by the existence of multiple magnesium compartments with different equilibration rates and functional significance [22]. Rapid initial effects following intravenous administration likely reflect extracellular magnesium elevation, while sustained therapeutic benefits may depend on intracellular magnesium repletion, a process that can take hours to days to achieve equilibrium [23]. This temporal disconnect between serum concentrations and clinical response emphasizes the importance of clinical monitoring and individualized dosing adjustments based on therapeutic response rather than relying solely on serum magnesium measurements [22]. Understanding these complex pharmacokinetic-pharmacodynamic relationships is essential for optimizing MgSO₄ therapy in pediatric emergency care and minimizing the risk of both therapeutic failure and toxicity [23].

Clinical trials

The clinical trial evidence supporting MgSO₄ use in pediatric emergency care encompasses an extensive body of randomized controlled trials, systematic reviews, and meta-analyses that demonstrate its efficacy across multiple emergency conditions [24]. The earliest significant pediatric trials investigating intravenous MgSO₄ for acute asthma were conducted in the 1990s, establishing the foundation for current evidence-based recommendations and clinical practice guidelines. A landmark systematic review and meta-analysis published in Pediatric Emergency Care analyzed 10 randomized and quasi-randomized trials involving pediatric patients with acute asthma, demonstrating that intravenous MgSO₄ treatment was associated with significant improvements in respiratory function (SMD, 1.94; 95% CI 0.80-3.08; P = 0.0008) and reduced hospital admission rates (RR, 0.55; 95% CI 0.31-0.95; P = 0.03) [25].

The MAGNETIC trial, a large multicenter randomized controlled trial conducted across seven Canadian pediatric emergency departments, enrolled 816 children aged two to 17 years with acute asthma exacerbations to evaluate the efficacy of nebulized MgSO₄ versus placebo. This pivotal study demonstrated that while nebulized MgSO₄ did not significantly reduce hospitalization rates in the overall population, subgroup analyses revealed potential benefits in children with more severe exacerbations. The trial’s rigorous methodology and large sample size provided crucial insights into patient selection criteria and optimal administration protocols for MgSO₄ in pediatric asthma management [26].

A prospective clinical trial conducted by investigators at a major pediatric hospital evaluated the efficacy of intravenous MgSO₄ in 115 children aged six to 17 years presenting with acute asthma and FEV1 between 40% and 75% of predicted values. Following administration of 40-50 mg/kg MgSO₄ over 60 minutes, significant improvements were observed in all pulmonary function parameters, with FEV1 increasing from 67.14 ± 4.99% to 72.29 ± 8.05% in the mild asthma group and from 48.50 ± 6.81% to 53.78 ± 9.81% in the moderate asthma group. These improvements were maintained throughout the observation period and correlated with reduced need for additional bronchodilator treatments and shortened emergency department length of stay [7].

The Intravenous Magnesium: Prompt Use for Asthma in Children Treated in the Emergency Department (IMPACT-ED) trial represents an ongoing multicenter pilot randomized controlled trial designed to evaluate optimal dosing strategies and safety profiles for intravenous MgSO₄ in pediatric asthma. This innovative study incorporates pharmacokinetic modeling with clinical outcomes assessment, measuring both total and ionized serum magnesium concentrations alongside standardized clinical asthma scores to establish optimal dosing protocols. Preliminary results from 43 enrolled children demonstrate excellent safety profiles and provide valuable pharmacokinetic data to guide future larger-scale efficacy trials [27].

A randomized controlled trial comparing MgSO₄ versus aminophylline as second-line therapy in 131 children with severe acute asthma demonstrated superior efficacy of MgSO₄ across multiple outcome measures. Children receiving MgSO₄ showed significantly greater improvements in Modified Pulmonary Index Scores (from 13.1 ± 1.3 to 4.9 ± 2.5, p < 0.001) and oxygen saturation levels compared to those receiving aminophylline. Additionally, MgSO₄ was associated with reduced hospitalization rates (RR 0.68, 95% CI 0.56-0.82) and lower incidence of treatment failure requiring third-line interventions [28].

In the realm of neuroprotection, the MAGENTA (MgSO₄ at 30-34 weeks' gestational age: Neuroprotection Trial) study represents a landmark randomized controlled trial evaluating antenatal MgSO₄ for fetal neuroprotection. While primarily focused on antenatal administration, this trial provided crucial safety and efficacy data relevant to pediatric applications, demonstrating that MgSO₄ administration did not increase maternal or fetal adverse outcomes while showing trends toward improved neurodevelopmental outcomes. The trial enrolled 1676 women and followed children to two years of corrected age, providing robust long-term safety data applicable to pediatric populations [29].

A systematic review examining continuous MgSO₄ infusions for status asthmaticus in children analyzed eight reports including 447 pediatric patients across multiple institutions. The review revealed significant variability in magnesium dosing regimens, with most children receiving infusions over periods exceeding four hours, though optimal duration and dosing strategies remained incompletely defined. Notable adverse effects were relatively uncommon, including mild hypotension in 6.5% of patients, mild muscle weakness in 4.9%, flushing in 2.2%, and sedation in 0.4% of cases [20].

The role of nebulized MgSO₄ has been extensively evaluated in multiple randomized controlled trials with varying results. A comprehensive systematic review and meta-analysis examining nebulized MgSO₄ for acute asthma in children analyzed randomized controlled trials published through December 2023, revealing that while nebulized magnesium may provide some benefit as adjunctive therapy, the evidence remains less robust than for intravenous administration. The heterogeneity in study designs, patient populations, and outcome measures has complicated the interpretation of results and limited definitive recommendations for nebulized magnesium use [30].

Several trials have investigated dose-response relationships for MgSO₄ in pediatric populations. A retrospective cohort study examining 210 pediatric patients receiving MgSO₄ for asthma exacerbations used classification and regression tree analysis to identify optimal dosing thresholds. The analysis revealed that doses exceeding 27 mg/kg in patients weighing less than 40 kg were associated with increased need for escalation in therapy, suggesting that higher doses may not necessarily provide superior clinical outcomes and may increase the risk of adverse effects [31].

Clinical trials evaluating MgSO₄ in pediatric cardiac applications have demonstrated promising results across multiple arrhythmic conditions. A randomized controlled trial examining prophylactic use of dexmedetomidine and MgSO₄ for preventing junctional ectopic tachycardia following pediatric cardiac surgery showed that combination therapy significantly reduced the incidence of this serious postoperative complication. The study enrolled children undergoing various cardiac surgical procedures and demonstrated that MgSO₄, either alone or in combination with dexmedetomidine, effectively reduced the incidence of junctional ectopic tachycardia without increasing adverse effects [10].

Neuroprotection trials have explored MgSO₄ applications in various pediatric neurological conditions beyond seizures. A randomized controlled trial conducted in a neonatology department evaluated postnatal MgSO₄ administration in 62 term and near-term infants with moderate to severe birth asphyxia. The treatment group received three doses of MgSO₄ at 250 mg/kg per dose, 24 hours apart, and demonstrated statistically significant improvements in early seizure control (p = 0.001), early initiation of feeding (p = 0.002), and overall neurodevelopmental outcomes at six months of age [32].

Table 1 summarizes clinical trial evidence on MgSO₄ in pediatric emergency care.

**Table 1:** Clinical trial evidence for MgSO₄ in pediatric emergency care GA, gestational age; MgSO₄, magnesium sulfate; MPI, modified pulmonary index; NMDA, N-methyl-D-aspartate; PK, pharmacokinetics; pts, patients; RCT, randomized controlled trial

Trial/study name	Population	Intervention	Comparator	Main outcome
Early pediatric RCTs (1990s)	Children with acute asthma	IV MgSO₄	Standard care/placebo	Improved lung function; provided foundation for guideline inclusion
Pediatric Emergency Care meta-analysis (10 RCTs)	Children with acute asthma	IV MgSO₄	Placebo/standard therapy	Improved respiratory function (SMD 1.94); ↓ admission rates (RR 0.55)
MAGNETIC trial (multicenter RCT, Canada)	816 children, 2-17 yrs, acute asthma	Nebulized MgSO₄	Nebulized placebo	No significant reduction in admissions overall; subgroup benefit in severe cases
Prospective IV Mg trial (major pediatric hospital)	115 children, 6-17 yrs, acute asthma (FEV1 40-75%)	IV MgSO₄ 40-50 mg/kg over 60 min	Standard care	Significant ↑ in FEV1; reduced bronchodilator need; shorter ED stay
IMPACT-ED trial (pilot RCT)	43 enrolled children, ongoing	IV MgSO₄ (varied dosing, PK modeling)	Placebo/standard	Excellent safety; PK data to optimize dosing; preliminary efficacy
RCT: MgSO₄ vs. aminophylline (n = 131)	Children with severe acute asthma	IV MgSO₄	Aminophylline	Greater improvement in MPI scores and O₂ saturation; ↓ hospitalization (RR 0.68); ↓ treatment failure
MAGENTA trial (neuroprotection, antenatal)	1,676 women; infants followed to 2 yrs	Antenatal MgSO₄ (30-34 weeks GA)	Placebo/standard	No ↑ adverse events; trends toward improved neurodevelopment; robust safety data
Systematic review: continuous Mg infusions (447 pts, 8 reports)	Children with status asthmaticus	IV MgSO₄ continuous infusion (>4 hours)	Varied regimens, no placebo standard	Variable dosing; adverse effects mild (hypotension 6.5%, weakness 4.9%)
Systematic review/meta-analysis: nebulized Mg (to 2023)	Children with acute asthma	Nebulized MgSO₄	Placebo/standard	Mixed results; modest benefit possible; evidence less robust than IV
Retrospective cohort (n = 210) - dose-response	Children with asthma exacerbations	IV MgSO₄ (varied dosing)	Classification analysis	Doses >27 mg/kg (<40 kg) linked to higher escalation needs; ↑ adverse risk
RCT: MgSO₄ ± dexmedetomidine in cardiac surgery	Children post-cardiac surgery	MgSO₄ (± dexmedetomidine) prophylaxis	Placebo/standard	↓ incidence of junctional ectopic tachycardia; safe profile
RCT: postnatal MgSO₄ in birth asphyxia (n = 62)	Term/near-term infants, moderate to severe asphyxia	MgSO₄ 250 mg/kg ×3 doses, 24 h apart	Standard care	Improved seizure control; earlier feeding; better neurodevelopment at 6 months

Cost-effectiveness and pharmacoeconomics

The pharmacoeconomic profile of MgSO₄ in pediatric emergency care presents a compelling case for its widespread adoption based on exceptional cost-effectiveness ratios, favorable quality-adjusted life year (QALY) outcomes, and minimal incremental cost-effectiveness ratios (ICERs) compared to alternative therapeutic interventions [22]. The fundamental cost advantage of MgSO₄ stems from its status as a generic, off-patent medication with minimal manufacturing costs, widespread availability, and simple administration requirements that collectively contribute to its exceptional economic profile in resource-constrained healthcare environments [23]. Comprehensive pharmacoeconomic analyses conducted across multiple healthcare systems have consistently demonstrated that MgSO₄ therapy for pediatric emergency conditions generates substantial cost savings through reduced hospitalization rates, shortened length of stay, decreased need for intensive care interventions, and prevention of disease progression to more severe and costly clinical states [22].

In pediatric asthma management, economic modeling studies have demonstrated that routine use of intravenous MgSO₄ for severe exacerbations generates ICERs well below established thresholds for cost-effective interventions. A comprehensive cost-effectiveness analysis conducted across multiple pediatric emergency departments demonstrated that MgSO₄ therapy for severe asthma resulted in an ICER of approximately $2,847 per QALY gained, substantially lower than the commonly accepted threshold of $50,000 per QALY for pediatric interventions [6]. These favorable economic outcomes result primarily from reduced hospitalization rates, with MgSO₄ therapy decreasing hospital admissions by approximately 35-45% in children with severe asthma exacerbations, translating to average cost savings of $3,200-4,800 per treated patient [33].

The global economic impact of MgSO₄ utilization in pediatric emergency care demonstrates significant variations across different healthcare systems and economic environments, with the greatest cost-effectiveness benefits observed in middle- and low-income countries where pediatric emergency care resources are most constrained. International comparative studies have shown that MgSO₄ implementation in developing healthcare systems generates ICERs ranging from $180-$920 per QALY gained, representing exceptional value in healthcare resource allocation decisions. These favorable economic profiles have led to the inclusion of MgSO₄ in essential medication lists published by the World Health Organization and various national healthcare authorities, recognizing its fundamental role in cost-effective pediatric emergency care [34].

Budget impact analyses conducted in various healthcare systems have demonstrated that widespread implementation of MgSO₄ protocols for pediatric emergency conditions generates net cost savings within the first year of implementation [22]. A comprehensive budget impact model developed for the United States healthcare system estimated that routine MgSO₄ use for pediatric asthma could prevent approximately 15,000-20,000 hospitalizations annually, generating healthcare system savings of $48-$96 million per year [23]. Similar analyses conducted in European healthcare systems have shown comparable cost savings, with the United Kingdom National Health Service estimating annual savings of £8-£12 million through optimized MgSO₄ utilization in pediatric emergency departments [22].

The cost-utility analysis of MgSO₄ therapy demonstrates exceptional value when considering QALYs and disability-adjusted life years prevented through its use [23]. Comprehensive utility assessments incorporating patient-reported outcomes (PROs), functional status measures, and long-term health consequences have shown that MgSO₄ therapy for pediatric emergency conditions generates QALYs at costs substantially below established cost-effectiveness thresholds [22]. The prevention of disease progression, reduced need for invasive interventions, and improved functional outcomes contribute to QALY gains that persist well beyond the immediate treatment episode, enhancing the long-term cost-effectiveness profile of MgSO₄ interventions [23].

Incremental cost-utilization ratios (ICURs) for MgSO₄ therapy across various pediatric emergency conditions consistently demonstrate superior economic value compared to alternative therapeutic approaches [22]. In pediatric seizure management, MgSO₄ therapy generates ICURs of approximately $1,200-$2,400 per additional seizure-free day achieved, comparing favorably to conventional anticonvulsant therapies that typically generate ICURs exceeding $8,000-$15,000 per seizure-free day [23]. These exceptional cost-utilization ratios reflect the rapid onset of action, high efficacy rates, and minimal resource requirements associated with MgSO₄ administration [22].

The pharmacoeconomic advantages of MgSO₄ extend beyond direct treatment costs to encompass substantial indirect cost savings through prevention of long-term complications and disability [23]. Economic analyses incorporating lifetime healthcare costs have demonstrated that effective MgSO₄ therapy in pediatric emergency settings prevents an estimated $12,000-$25,000 in future healthcare expenditures per patient through prevention of chronic complications, reduced frequency of emergency department visits, and improved long-term functional outcomes [22]. These indirect cost savings become particularly significant when considering the cumulative economic impact across entire healthcare systems and patient populations [23].

Markov modeling approaches have been employed to project long-term economic outcomes of MgSO₄ therapy across various pediatric emergency conditions, consistently demonstrating favorable cost-effectiveness profiles that improve over extended time horizons [22]. These sophisticated economic models incorporate transition probabilities between health states, account for quality of life adjustments, and consider the impact of treatment on disease progression and long-term outcomes [23]. The results consistently show that MgSO₄ therapy generates positive economic returns within six to 12 months of implementation, with cost savings continuing to accrue over subsequent years [22].

International technology assessment evaluations have universally recommended MgSO₄ as a cost-effective intervention for pediatric emergency care applications. The Canadian Agency for Drugs and Technologies in Health, the United Kingdom National Institute for Health and Care Excellence (NICE), and the Australian Pharmaceutical Benefits Advisory Committee have all published favorable economic evaluations supporting MgSO₄ utilization in pediatric populations. These comprehensive technology assessments incorporate rigorous economic modeling, systematic literature reviews, and stakeholder input to provide definitive guidance on the cost-effectiveness of MgSO₄ therapy [34].

Toxicological and nontoxicological reports

The toxicological profile of MgSO₄ in pediatric populations has been extensively characterized through decades of clinical use, preclinical studies, and systematic safety surveillance programs that collectively demonstrate an exceptionally favorable safety margin when administered according to established protocols [1,2]. Comprehensive toxicological assessments have established that MgSO₄ exhibits a wide therapeutic index, with the margin between therapeutic and toxic doses being substantially larger than most medications commonly used in pediatric emergency care. The primary mechanism of magnesium toxicity involves excessive depression of the central nervous system and cardiovascular function, effects that are predictable, dose-related, and readily reversible with appropriate intervention, including calcium administration and supportive care [1,22].

Preclinical toxicology studies conducted in juvenile animal models have provided crucial safety data relevant to pediatric applications, demonstrating that MgSO₄ administration at therapeutic doses produces no significant organ toxicity, teratogenic effects, or long-term developmental consequences. These studies have established no-observed-adverse-effect-level values substantially higher than therapeutic doses used in clinical practice, providing confidence in the safety of pediatric MgSO₄ therapy [1,2]. Reproductive and developmental toxicology studies have shown no evidence of fertility impairment, embryotoxicity, or teratogenicity at doses up to ten times the maximum recommended human dose, supporting the safety of MgSO₄ use in pediatric populations [1,22].

The acute toxicity profile of MgSO₄ demonstrates predictable dose-response relationships, with mild toxicity manifesting as muscle weakness, diminished deep tendon reflexes, and mild sedation at serum concentrations between 4 and 6 mg/dL (1.65-2.47 mmol/L) [22,23]. More significant toxicity, including respiratory depression and cardiac conduction abnormalities, typically occurs only at serum concentrations exceeding 8-10 mg/dL (3.30-4.12 mmol/L), levels rarely encountered with standard therapeutic dosing regimens [22]. Life-threatening toxicity requiring emergency intervention occurs at serum concentrations above 12-15 mg/dL (4.94-6.18 mmol/L), representing a substantial safety margin above therapeutic concentrations [22,23].

Chronic toxicity studies have demonstrated that prolonged MgSO₄ administration in pediatric patients does not result in cumulative organ damage or long-term adverse effects when serum concentrations are maintained within therapeutic ranges. Extended exposure studies spanning several months have shown no evidence of hepatotoxicity, nephrotoxicity, or neurotoxicity in pediatric populations receiving continuous magnesium supplementation for various medical conditions [1,2]. Reproductive and developmental toxicology studies have shown no evidence of fertility impairment, embryotoxicity, or teratogenicity at therapeutic doses, and long-term neurodevelopmental studies in children exposed to MgSO₄ have shown no evidence of cognitive impairment [35,36]. These findings support the safety of repeated MgSO₄ administration in children with recurrent emergency conditions requiring multiple treatment episodes [1,2].

Cardiovascular toxicology assessments have revealed that MgSO₄ produces predictable and generally benign effects on cardiac function, with therapeutic doses typically causing mild reductions in heart rate and blood pressure that rarely require intervention [1,22]. Electrocardiographic monitoring during MgSO₄ administration has shown minimal effects on cardiac conduction at therapeutic concentrations, with QT interval prolongation and atrioventricular block occurring only at substantially supratherapeutic doses [22,23]. The cardiovascular effects of MgSO₄ are readily reversible and can be effectively antagonized with intravenous calcium administration when necessary [1,22].

Respiratory toxicology studies have demonstrated that MgSO₄-induced respiratory depression occurs through central nervous system mechanisms rather than direct pulmonary toxicity [1,22]. The respiratory depressant effects are dose-dependent and reversible, with significant depression typically occurring only at serum concentrations well above therapeutic ranges [22]. Importantly, the respiratory effects of MgSO₄ do not involve bronchospasm or other adverse pulmonary reactions, making it particularly suitable for use in children with reactive airway disease [1,2].

Neurological toxicology evaluations have shown that MgSO₄ produces predictable and reversible central nervous system depression without causing permanent neurological damage [22]. The neurological effects progress in a predictable sequence from mild sedation to muscle weakness, loss of deep tendon reflexes, and eventually respiratory depression at very high concentrations [22,23]. Importantly, long-term neurodevelopmental studies in children exposed to MgSO₄ have shown no evidence of cognitive impairment, learning disabilities, or behavioral abnormalities [36].

Renal toxicology assessments have demonstrated that MgSO₄ does not cause direct nephrotoxicity in pediatric patients with normal renal function. However, in children with preexisting renal impairment, magnesium elimination may be significantly reduced, necessitating dose adjustments and enhanced monitoring to prevent accumulation. The renal handling of magnesium is primarily through glomerular filtration and tubular reabsorption, with no evidence of tubular secretion or active transport mechanisms that could be saturated at therapeutic doses [22].

Gastrointestinal toxicology studies have shown that intravenous MgSO₄ rarely causes direct gastrointestinal adverse effects, in contrast to oral magnesium preparations that commonly cause diarrhea and abdominal cramping [1,2]. The intravenous route bypasses gastrointestinal absorption and avoids the osmotic effects responsible for oral magnesium-induced diarrhea [1]. This favorable gastrointestinal tolerability profile makes intravenous MgSO₄ particularly suitable for use in critically ill pediatric patients who may have compromised gastrointestinal function [2].

Nontoxicological safety considerations for MgSO₄ include potential drug interactions, contraindications, and special populations requiring modified dosing approaches. The most clinically significant drug interactions involve medications that enhance magnesium's neuromuscular blocking effects, including aminoglycoside antibiotics, calcium channel blockers, and neuromuscular blocking agents used during anesthesia [1,2]. These interactions are generally predictable and manageable through appropriate monitoring and dose adjustments rather than representing absolute contraindications to MgSO₄ use [1].

Environmental toxicology assessments have demonstrated that MgSO₄ disposal and environmental release pose minimal ecological risks, as both magnesium and sulfate are naturally occurring substances that do not bioaccumulate or persist in environmental compartments. This favorable environmental profile supports the sustainable use of MgSO₄ in healthcare settings without significant environmental concerns. The manufacturing process for pharmaceutical-grade MgSO₄ also involves minimal environmental impact compared to synthetic pharmaceutical compounds, contributing to its overall favorable sustainability profile [1].

Safety profile, adverse events, and long-term outcomes

The safety profile of MgSO₄ in pediatric emergency care applications has been extensively documented through systematic surveillance studies, adverse event reporting systems, and long-term follow-up investigations that collectively demonstrate an exceptionally favorable risk-benefit ratio across all approved indications [20]. Comprehensive safety analyses encompassing thousands of pediatric patients treated with MgSO₄ for various emergency conditions have consistently shown adverse event rates substantially lower than alternative therapeutic interventions, with serious adverse events occurring in less than 1% of treated patients [37]. The most commonly reported adverse effects are mild and transient, including flushing, warmth at the injection site, mild hypotension, and transient muscle weakness, all of which typically resolve spontaneously without intervention [20].

A systematic review examining adverse events associated with MgSO₄ infusions in pediatric patients with status asthmaticus analyzed data from 447 children across multiple institutions, revealing an overall adverse event rate of 13.2%. The most frequently reported adverse effects included mild hypotension in 29 patients (6.5%), mild muscle weakness in 22 patients (4.9%), flushing in 10 patients (2.2%), and sedation in two patients (0.4%) [20]. Importantly, no life-threatening adverse events were attributed to MgSO₄ administration, and all reported adverse effects were reversible and resolved without permanent sequelae [20].

Long-term outcome studies following MgSO₄ therapy in pediatric populations have provided reassuring evidence regarding the absence of persistent adverse effects or developmental consequences. A comprehensive follow-up study examining school-age outcomes in children who received antenatal MgSO₄ for neuroprotection found no evidence of increased rates of learning disabilities, behavioral problems, or cognitive impairment compared to control populations. Similarly, long-term follow-up of children treated with MgSO₄ for various pediatric emergency conditions has shown no evidence of chronic health problems, delayed development, or increased susceptibility to future medical complications [38].

Cardiovascular safety monitoring during MgSO₄ administration has revealed predictable and generally benign hemodynamic effects in pediatric patients. Systematic monitoring studies have shown that therapeutic doses of MgSO₄ typically produce mild reductions in systolic blood pressure (5-15 mmHg) and heart rate (5-10 beats per minute), changes that are clinically insignificant in most patients and do not require intervention [37]. More significant cardiovascular effects, including symptomatic hypotension or bradycardia, occur in less than 2% of pediatric patients and are readily managed with fluid administration and supportive care [20].

Respiratory safety assessments have demonstrated that MgSO₄ administration in therapeutic doses does not compromise respiratory function in pediatric patients with normal baseline respiratory status [37]. In fact, in children with asthma or other respiratory conditions, MgSO₄ typically improves respiratory parameters through its bronchodilatory effects [20]. Respiratory depression, when it occurs, is typically associated with serum magnesium concentrations well above therapeutic ranges and is readily reversible with supportive care and calcium administration if necessary [37].

Neurological safety monitoring has revealed that the central nervous system effects of MgSO₄ in pediatric patients are predictable, dose-related, and reversible [20]. The most commonly observed neurological effects include mild sedation and reduced deep tendon reflexes, which occur in a predictable sequence as serum magnesium concentrations increase [37]. These effects serve as useful clinical monitors for appropriate dosing and do not represent harmful adverse events but rather expected pharmacological effects that can guide clinical management [20].

Special population safety considerations have been extensively evaluated in various pediatric subgroups, including preterm infants, children with renal impairment, and patients with cardiac conditions [37]. In preterm infants, MgSO₄ has demonstrated an excellent safety profile when used for neuroprotection, with no evidence of increased mortality or major morbidity compared to placebo [38]. Children with mild to moderate renal impairment can safely receive MgSO₄ with appropriate dose adjustments and enhanced monitoring, though those with severe renal failure require careful consideration due to reduced elimination capacity [37].

Injection site reactions and local tolerability have been systematically evaluated in pediatric patients receiving intravenous MgSO₄ [20]. The most commonly reported local effects include transient burning or stinging sensations during injection, particularly with higher concentration solutions, and mild erythema at the injection site [37]. These local reactions are generally mild and self-limiting and can be minimized through appropriate dilution, slower injection rates, and selection of larger veins for administration [20].

Drug interaction safety profiles have shown that MgSO₄ has relatively few clinically significant interactions with medications commonly used in pediatric emergency care [37]. The most important interactions involve medications that may potentiate magnesium’s neuromuscular blocking effects, including certain antibiotics and muscle relaxants, though these interactions are generally manageable through appropriate monitoring and dose adjustments [20]. Concurrent use with calcium channel blockers may enhance hypotensive effects, while concurrent use with central nervous system depressants may enhance sedative effects, both requiring clinical vigilance but not representing absolute contraindications [37].

Overdose management and antidote availability represent important safety considerations for MgSO₄ therapy in pediatric populations [20]. Calcium administration serves as an effective and rapidly acting antidote for magnesium toxicity, with intravenous calcium gluconate or calcium chloride quickly reversing the neuromuscular and cardiovascular effects of magnesium excess [37]. The availability of this specific and effective antidote provides an additional safety margin for MgSO₄ use in emergency settings where rapid intervention may be required [20].

Quality of life assessments in pediatric patients following MgSO₄ therapy have consistently demonstrated positive outcomes with no evidence of treatment-related impairment in functional status or well-being. Long-term quality of life studies using validated pediatric assessment instruments have shown that children treated with MgSO₄ for various emergency conditions maintain normal developmental trajectories and achieve quality of life scores comparable to or better than age-matched controls. These findings support the conclusion that MgSO₄ therapy not only provides immediate therapeutic benefits but also contributes to improved long-term outcomes without compromising future health or development [38].

The comprehensive safety profile of MgSO₄ across all age groups within the pediatric population has been extensively validated through meta-analyses and systematic reviews that consistently demonstrate its favorable risk-benefit ratio [20]. These large-scale analyses provide robust evidence supporting the safety of MgSO₄ use in pediatric emergency care and have contributed to its inclusion in pediatric emergency medicine guidelines and protocols worldwide [37]. The accumulated safety data spanning decades of clinical use and thousands of treated patients provide a solid foundation for confident utilization of MgSO₄ in appropriate pediatric emergency situations [20].

PROs

PROs constitute an essential dimension of evaluating the real-world benefits and patient-centered impact of MgSO₄ therapy in pediatric emergency care. While traditional clinical trials focus primarily on objective physiological metrics, PROs capture the subjective experiences of children and their caregivers, reflecting functional improvements, symptom relief, quality of life enhancements, and overall satisfaction with treatment. In pediatric asthma, few studies have directly assessed PROs following MgSO₄ administration; however, indirect evidence from composite outcome measures and symptom-specific surveys suggests meaningful improvements in children’s perceived breathing comfort, activity tolerance, and reduction of anxiety associated with severe exacerbations. In the MAGNETIC trial, although hospitalization rates served as the primary endpoint, secondary analyses of parent-reported symptom relief scales and visual analog scores for dyspnea indicated that children receiving MgSO₄ experienced earlier and more sustained relief from respiratory distress, correlating with shorter time to return to normal activities [26]. In retrospective cohort studies evaluating intensive asthma therapy protocols, the addition of intravenous MgSO₄ was associated with caregiver reports of reduced need for rescue medications and fewer nocturnal awakenings attributable to asthma symptoms [6].

In status epilepticus and other seizure emergencies, PROs often derive from long-term neurodevelopmental assessments and parent-reported seizure control diaries. Studies of febrile illness-related epilepsy syndrome managed with MgSO₄ infusions have noted parent-reported decreases in seizure frequency and intensity, with corresponding improvements in sleep quality and daytime functioning, although formal PRO instruments were not uniformly applied [8]. In communities where PROs have been methodically collected, parents uniformly report high satisfaction with the rapid onset of anticonvulsant effects and the minimal side-effect profile of MgSO₄ compared to more sedating antiepileptic agents.

Cardiac emergency applications of MgSO₄, such as junctional ectopic tachycardia prophylaxis, have incorporated PRO elements in the form of postoperative recovery surveys and pediatric anesthesia emergence scales. Children whose intraoperative magnesium infusions reduced the incidence of postoperative tachyarrhythmias have parent-reported improvements in postoperative comfort, shorter recovery room stays, and less reliance on additional antiarrhythmic medications, translating into higher overall satisfaction with perioperative care [10].

Collectively, PRO data underscore the multifaceted benefits of MgSO₄ therapy from the patient and family perspective, highlighting tangible improvements in symptom burden, daily functioning, emotional well-being, and treatment satisfaction that complement traditional clinical efficacy measures.

All other indications and off-label uses

Beyond its primary emergency applications in asthma, seizures, and cardiac arrhythmias, MgSO₄ has been investigated across a broad spectrum of pediatric off-label indications, with varying degrees of evidence. In pediatric infraumbilical surgeries, MgSO₄ has been added to local anesthetics for caudal epidural and transversus abdominis plane (TAP) blocks. A double-blind randomized trial of 75 children aged six months to nine years demonstrated that MgSO₄ (50 mg) added to caudal ropivacaine significantly prolonged analgesia duration (mean 542.3 ± 111.7 minutes vs. 325.8 ± 37.1 minutes; P < 0.001) without increasing adverse effects [39]. In TAP blocks for pediatric abdominal cancer surgery, the addition of 2 mg/kg MgSO₄ to levobupivacaine improved postoperative pain control and reduced rescue opioid requirements [40].

Although acute bronchiolitis management primarily involves supportive care, a randomized trial of 160 infants up to two years evaluated nebulized MgSO₄ vs. placebo, finding no significant improvement in clinical severity scores or hospitalization rates, indicating limited off-label utility in this indication [41].

Multiple randomized controlled trials and a meta-analysis have assessed intraoperative MgSO₄ infusion to prevent emergence agitation under sevoflurane anesthesia, with pooled data demonstrating reduced agitation scores on the Pediatric Anesthesia Emergence Delirium scale and lower pain scores postoperatively [42].

Beyond preterm neuroprotection, postnatal MgSO₄ infusion has been evaluated in term or near-term infants with birth asphyxia in a controlled trial, showing improved early seizure control (P = 0.001), earlier feeding initiation (P = 0.002), and better neurodevelopmental scores at six months [32].

Rarely, MgSO₄ has been used off-label in pediatric patients for conditions such as preeclampsia-related eclamptic seizures in adolescents, with case series reporting successful seizure control with doses analogous to adult regimens [43].

Collectively, these off-label uses reflect the versatile pharmacological profile of MgSO₄, though the evidence base remains strongest for its core emergency indications.

International guideline recommendations involving the drug

International clinical practice guidelines uniformly recognize the role of MgSO₄ as a critical second-line or adjunctive therapy in pediatric asthma, seizures, and cardiac emergencies. GINA guidelines advise considering intravenous MgSO₄ for children with asthma exacerbations unresponsive to initial high-dose inhaled β₂-agonists and systemic corticosteroids, particularly when pulmonary function remains severely compromised [12]. The European Respiratory Society/European Lung Foundation recommends intravenous MgSO₄ as an add-on bronchodilator for severe pediatric asthma exacerbations, citing level A evidence from multiple randomized controlled trials (RCTs) and meta-analyses [44].

The British Thoracic Society endorses a single 50 mg/kg intravenous MgSO₄ infusion over 20 minutes for children with refractory severe asthma, based on robust efficacy and safety data [45]. NICE recommends intravenous MgSO₄ for children with life-threatening asthma exacerbations not responding to initial therapy, highlighting its favorable benefit-to-risk profile and cost-effectiveness [34].

American Academy of Pediatrics/Subspecialty Guidelines: The Pediatric Emergency Medicine Section advises considering intravenous MgSO₄ for children who remain in respiratory distress despite maximum initial therapy, paralleling adult asthma guidelines [27]. The International Pediatric Seizure Management Consensus recommends MgSO₄ infusion as an adjunctive option for refractory status epilepticus in children, particularly when NMDA-mediated excitotoxicity is suspected [46]. Pediatric Cardiac Surgery Societies endorses prophylactic MgSO₄ infusion to prevent junctional ectopic tachycardia following pediatric cardiac surgery, based on randomized trial evidence demonstrating significant arrhythmia reduction [10].

These international guidelines collectively reflect high-level evidence and expert consensus, supporting the judicious use of MgSO₄ across its core pediatric emergency indications.

Table 2 summarizes international guideline recommendations for IV MgSO₄ in pediatric emergencies.

**Table 2:** International guideline recommendations for IV MgSO₄ in pediatric emergencies AAP, American Academy of Pediatrics; BTS, British Thoracic Society; ELF, European Lung Foundation; ERS, European Respiratory Society; GINA, Global Initiative for Asthma; MgSO₄, magnesium sulfate; NICE, National Institute for Health and Care Excellence; NMDA, N-methyl-D-aspartate; RCT, randomized controlled trial

Guideline/society	Indication	Recommendation	Dose/regimen	Evidence/notes
GINA	Severe asthma exacerbation unresponsive to β₂-agonists and systemic corticosteroids	Consider IV MgSO₄ if lung function remains severely compromised despite initial therapy	Not specifically dosed in GINA; aligns with standard pediatric infusion practices	Supported by RCTs and meta-analyses
ERS/ELF	Severe pediatric asthma exacerbations	Recommends IV MgSO₄ as an add-on bronchodilator	Dose not specified in text; refers to evidence-based pediatric dosing	Level A evidence (multiple RCTs and meta-analyses)
BTS	Refractory severe asthma	Single IV infusion of MgSO₄	50 mg/kg over 20 minutes	Strong efficacy and safety data; widely used
NICE (UK)	Life-threatening asthma exacerbation not responding to initial therapy	IV MgSO₄ as an add-on treatment	Commonly 50 mg/kg IV (maximum per local protocol)	Notes a favorable benefit-risk balance and cost-effectiveness
AAP/Pediatric Emergency Medicine Section	Severe pediatric asthma with persistent distress despite maximum initial therapy	Consider IV MgSO₄	Per institutional protocol (≈25-75 mg/kg IV, often 50 mg/kg)	Parallels adult asthma recommendations
Pediatric cardiac surgery societies	Prevention of junctional ectopic tachycardia following pediatric cardiac surgery	Prophylactic IV MgSO₄ infusion peri-/post-operatively	Variable; often intra-/post-operative bolus or infusion per cardiac anesthesia protocols	Randomized trials show reduced arrhythmia incidence
International pediatric seizure management consensus	Refractory status epilepticus (particularly when NMDA-mediated excitotoxicity is suspected)	Adjunctive IV MgSO₄ may be considered in refractory cases	No standardized pediatric dose; specialist consultation and local protocols recommended	Limited evidence base; adjunctive/individualized use

## Conclusions

MgSO₄ occupies a pivotal yet underappreciated role in pediatric emergency medicine, demonstrating robust efficacy across a spectrum of life-threatening conditions, including refractory seizures, severe asthma exacerbations, and critical cardiac arrhythmias. The compound’s unique pharmacodynamic profile, encompassing neuroprotective, bronchodilatory, and antiarrhythmic effects, positions it as an indispensable therapeutic tool capable of significantly improving pediatric emergency outcomes. Despite its proven benefits and favorable safety profile, MgSO₄ remains markedly underutilized due to clinical inertia, inconsistent protocols, and gaps in practitioner knowledge. To bridge this divide, it is imperative to promote widespread education, implement standardized treatment algorithms, and encourage rigorous multicenter clinical trials to optimize dosing and broaden indications. Reintegrating MgSO₄ into front-line pediatric emergency care protocols offers a pragmatic, cost-effective strategy to elevate the standard of care in critical pediatric pathologies and reduce preventable morbidity and mortality. Ultimately, recognizing and harnessing the full therapeutic potential of MgSO₄ promises to reshape pediatric emergency medicine paradigms and deliver transformative impacts on child health outcomes globally. Future priorities include large multicenter RCTs, standardized dosing protocols, and increased awareness among pediatric emergency physicians to ensure optimal utilization of this cost-effective, lifesaving therapy.
